# Resolution of Acute Hydrocephalus and Migration of Neurocysticercosis Cyst with External Ventricular Drainage

**DOI:** 10.1155/2010/245259

**Published:** 2010-05-25

**Authors:** Abhineet Chowdhary, Taylor J. Abel, Patrik Gabikian, Gavin W. Britz

**Affiliations:** ^1^Department of Neurological Surgery, Harborview Medical Center, University of Washington School of Medicine, 401 Broadway, 5th Floor, Patricia Steele Building, Seattle, WA 98103, USA; ^2^Division of Neurosurgery, Duke University Medical Center, Duke University, Durham, NC 27708, USA

## Abstract

Neurocysticercosis is endemic in the developing world, but is becoming more common in the US due to immigration. A 24-year-old man presented with acute hydrocephalus and headaches, nausea, and vomiting. Head CT revealed a 3rd ventricular cyst and immunological studies were suggestive of neurocysticercosis. EVD placement resulted in migration of the cyst interiorly and superiorly with return of normal CSF flow by MRI and resolution of symptoms. Review of this condition is important given increasing incidence in the United States.

## 1. Introduction

Cysticercosis is a disease caused by the larval stage of the tapeworm, *taenia solium, *and has become an endemic disease in the non-Muslim countries in Latin America, Asia, and Africa. By some reports, 60%–90% of the cases of Cysticercosis have some CNS involvement [1]. In the United States alone, there are around 1000 new cases of neurocysticercosis a year [2, 3], which is likely to increase with immigration. We present a case of a neurocysticercosis cyst causing transient CSF flow obstruction secondary to a mobile cyst in the 3rd ventricle and review clinical course and imaging findings.

## 2. Case Report

**Figure 1:**
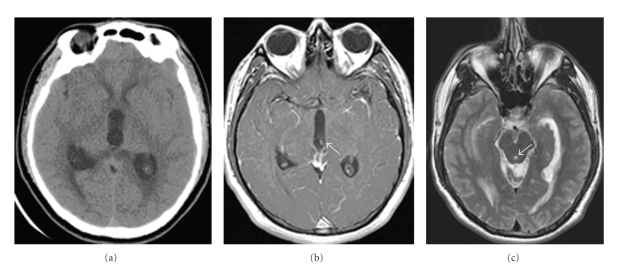
(a) CT head without contrast, (b) MRI T1 with contrast showing the cyst in the posterior third ventricle (arrow), and (c) MRI T2 showing altered flow through the Sylvian aqueduct (arrow).

**Figure 2:**
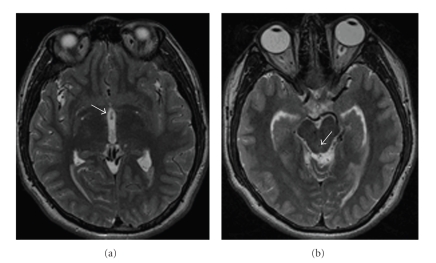
(a) MRI T2 showing migration of the cyst to the anterior third ventricle (arrow) and (b) MRI T2 demonstrating a flow void in the Sylvian aqueduct consistent with restoration of CSF flow (arrow). Of note, reduction in the size of the temporal horns is also appreciated.

This is a 24-year-old man who presented with three weeks of headache, which occasionally resolved with ibuprofen and acetaminophen. The day prior to admission, the patient had a severe headache associated with nausea and multiple bouts of emesis. The patient had increasing forgetfulness, as relayed by his mother, and, on the day of admission, experienced general malaise. He did not have any photophobia. He denied history of migraine or recent sick contacts. He emigrated from Mexico one year prior to presentation. There was no family history of migraine or headaches. The patient was alert and oriented and cranial nerves II-XII were intact. Vision was intact to gross examination. Initial head CT obtained in the ER to evaluate for headache showed ventriculomegaly suggestive of obstructive hydrocephalus with obstruction in the posterior third ventricle and an atypical appearance in the third ventricle region (Figure 1). An EVD was placed urgently to treat the obstructive hydrocephalus. MR imaging after EVD placement revealed migration of cyst interiorly and superiorly with resultant return of normal CSF flow by MRI (Figure 2). Migration of the cyst resulted in restoration of CSF flow and complete resolution of the patient's symptoms. 

**Figure 3:**
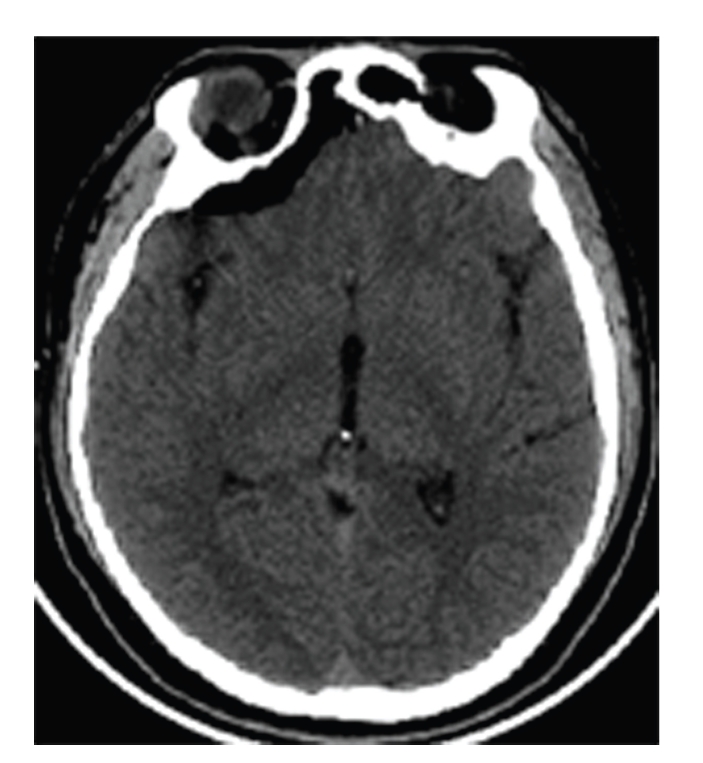
Head CT without contrast demonstrating complete removal of cyst.

The patient went to the OR where an endoscopic transcortical approach using frameless stereotaxy was used to remove the cyst from the brain. Postoperative brain MR revealed an absence of the cyst (Figure 3). The patient tolerated the procedure well and was eventually transferred to a rehab facility for a short inpatient stay. Postoperatively, the patient had complete resolution of his presenting symptoms. 

Pathology findings showed portions of a cyst wall with an outer cuticle, middle cellular layer, and innermost reticular layer. The cyst contains larval stage of parasite showing gut and scolex with muscular suckers and rostellum containing hooklets, suggestive of neurocysticercosis.

## 3. Discussion

Neurocysticercosis is becoming an increasing problem in the United States due to migration patterns, especially emigration from South and Central America. While both systemic and cranial infections can be asymptomatic, intraventricular neurocysticercosis can be quite deadly. Intraventricular cysts occur most commonly in the fourth ventricle because of gravity and CSF flow patterns, but cysts can be found in any part of the ventricles [4]. By physically obstructing the CSF pathway, intraventricular cysts may cause a noncommunicating hydrocephalus by mechanically obstructing CSF flow. 

Intraventricular neurocysticercosis has a risk of ependymitis in those treated with antihelminthics, thus necessitating surgical evaluation prior to medical treatment [5, 6]. Mobile cysts are well described in the phenomenon known as Bruns' Syndrome, which causes acute intermittent hydrocephalus [7]. It has been previously reported that a neurocysticercosis cyst had migrated from the lateral ventricle to the foramen of Monro causing sudden death [2]. Furthermore, another study examining intraventricular cysticercosis showed that 6 of 46 patients died from acute hydrocephalus after hospital admission [8]. 

Given the significant impact of cyst location, localization of neurocysticercosis cysts by neuroimaging is crucial for effective management of this condition. Neuroimaging findings are variable depending on the stage of the infection. During the vesicular stage, cysts and scolex are both imaged without enhancement [9]. In the colloidal vesicular stage, ring enhancement and edema are appreciated by both CT and MR imagings [9]. The granular nodular phase is characterized by decreased ring enhancement and edema, along with the calcification of cysts [9]. During the final involution stage, calcification is observed on CT and MR imagings as small areas of hypointensity [9]. 

Treatment options for intracranial cysts have included both medical as well as surgical therapies depending on location. Parenchymal cysts have historically been treated quite effectively with antihelminthics such as praziquantel and albendazole [10]. Infratentorial intraventricular cysts have been treated with open surgery for excision. And it has been advocated that supratentorial cysts, due to not only location but also the need to often treat hydrocephalus in these patients, be removed endoscopically [11]. The case presented here is an important reminder that intraventricular neurocysticercosis should be considered in the differential diagnosis of obstructive hydrocephalus with radiographic appearance of a cystic lesion in the third, aqueduct, or fourth ventricle.
